# Arsenic-induced sub-lethal stress reprograms human bronchial epithelial cells to CD61¯ cancer stem cells

**DOI:** 10.18632/oncotarget.1789

**Published:** 2013-02-19

**Authors:** Qingshan Chang, Bailing Chen, Chitra Thakur, Yongju Lu, Fei Chen

**Affiliations:** ^1^ Department of Pharmaceutical Sciences, Eugene Applebaum College of Pharmacy and Health Sciences, Wayne State University, Detroit, MI, USA

**Keywords:** cancer stem cells, arsenic, self-renewal, sub-lethal stress, JNK

## Abstract

In the present report, we demonstrate that sub-lethal stress induced by consecutive exposure to 0.25 μM arsenic (As^3+^) for six months can trigger reprogramming of the human bronchial epithelial cell (BEAS-2B) to form cancer stem cells (CSCs) without forced introduction of the stemness transcription factors. These CSCs formed from As^3+^-induced sub-lethal stress featured with an increased expression of the endogenous stemness genes, including Oct4, Sox2, Klf4, Myc, and others that are associated with the pluripotency and self-renewal of the CSCs. Flow cytometry analysis indicated that 90% of the CSC cells are CD61¯, whereas 100% of the parental cells are CD61^+^. These CD61¯ CSCs are highly tumorigenic and metastatic to the lung in xenotransplantation tests in NOD/SCID Il2rγ^−/−^ mice. Additional tests also revealed that the CD61¯ CSCs showed a significant decrease in the expression of the genes important for DNA repair and oxidative phosphorylation. To determine the clinical relevance of the above findings, we stratified human lung cancers based on the level of CD61 protein and found that CD61^low^ cancer correlates with poorer survival of the patients. Such a correlation was also observed in human breast cancer and ovarian cancer. Taken together, our findings suggest that in addition to the traditional approaches of enforced introduction of the exogenous stemness circuit transcription factors, sub-lethal stress induced by consecutive low dose As^3+^ is also able to convert non-stem cells to the CSCs.

## INTRODUCTION

Arsenic, especially the inorganic trivalent form (As^3+^), has been classified as a group I human carcinogen by the International Agency for Research on Cancer (IARC), the Environmental Protection Agency (EPA), and the Agency for Toxic Substances and Disease Registry (ATSDR) [1]. Substantial evidence from epidemiologic and experimental studies has clearly indicated that long-term exposure to As^3+^, either from drinking water contamination or air pollution, can cause cancers of the skin, lung, liver, kidney, prostate, and bladder [2]. Despite extensive studies and the implementation of new standards to reduce the levels of As^3+^ exposure, environmental As^3+^ exposure is still a major concern of public health in many areas of the world, such as areas in Bangladesh, China, Chile, Argentina, Australia, Mexico, Taiwan, Vietnam, West Bengal (India), and the southwestern regions of the United States of America. Globally, more than 100 million people are currently exposed to drinking water containing As^3+^ that is above the maximum contamination level of 10 ppb (0.13 μM, 10 μg/L) set by the World Health Organization (WHO) [3].

The mechanisms of how As^3+^ exposure causes human cancer have yet to be fully elucidated. Several reports demonstrated that As^3+^ is a poor mutagen in classic mutagenesis assays and indicated that the carcinogenic effect of As^3+^ is most likely due to its ability to induce oxidative stress responses that are linked to DNA damage, protein degradation, and gene transcription [2]. In addition, As^3+^ is able to induce the biogenesis of microRNA-190 (miR-190) that is responsible for the down-regulation of PHLPP. The down-regulation of PHLPP leads to the sustained activation of Akt, a protein kinase that is important for cell transformation and tumorigenic angiogenesis [4]. Furthermore, other recent studies showed that As^3+^ is able to activate a signaling cascade from JNK and Stat3 to Akt-mediated EZH2 phosphorylation, which may be linked to the epigenetic reprogramming of the genome and the malignant transformation of cells [5]. Finally, As^3+^ had been shown to be capable of converting normal stem cells into cancer stem cells (CSCs) in several experimental settings [6, 7].

CSCs represent a small reservoir of tumor cells that have the ability to self-renew and differentiate into diverse cancer cell progeny that form the bulk of tumors [8-10]. It is generally believed that CSCs are the major contributors to the sustained growth, heterogeneity, recurrence, metastasis, and therapeutic failure of tumors [11]. Although the first evidence for CSCs was documented in hematological malignancies, in which only a small subset of cancer cells were capable of forming new tumors when transplanted into immunodeficient mice [12, 13], emerging evidence suggests that CSCs are also responsible for the continued expansion of the malignant cell population in solid tumors of the brain, breast, liver, and prostate, among others. [14, 15]. The origin of CSCs is currently a subject of extensive debate. It is becoming evident that CSCs may be derived either from normal stem cells or progenitor cells, which acquired the features of malignant transformation in some types of cancer, or from terminally differentiated cancer cells or normal cells due to dedifferentiation in other types of tumors.

With most CSC studies focusing on the four Yamanaka factors (Oct4, Sox2, Klf4, and c-Myc, hereafter referred to as Myc), which are essential for the stemness of embryonic stem cells (ESCs), stem cells (SCs), induced pluripotent cells (iPSCs), and CSCs, the potential role of extracellular stimuli, such as sub-lethal stress induced by As^3+^, in CSC induction has been understudied, and its relevance to cancer development has remained obscure. In the present study, we demonstrate that As^3+^ is capable of reprogramming bronchial epithelial cells into CD61¯ CSCs without enforced expression of the Yamanaka factors. Furthermore, a consecutive low-dose As^3+^ treatment, which induced a sub-lethal stress of the cells, induces a sustained activation of JNK, leading to elevated expression of endogenous Myc, which may be critical for epithelial cell reprogramming and CSC formation.

## RESULTS

### Sub-lethal stress induced by consecutive low concentration As^3+^ treatment triggers malignant transformation

**Figure 1:**
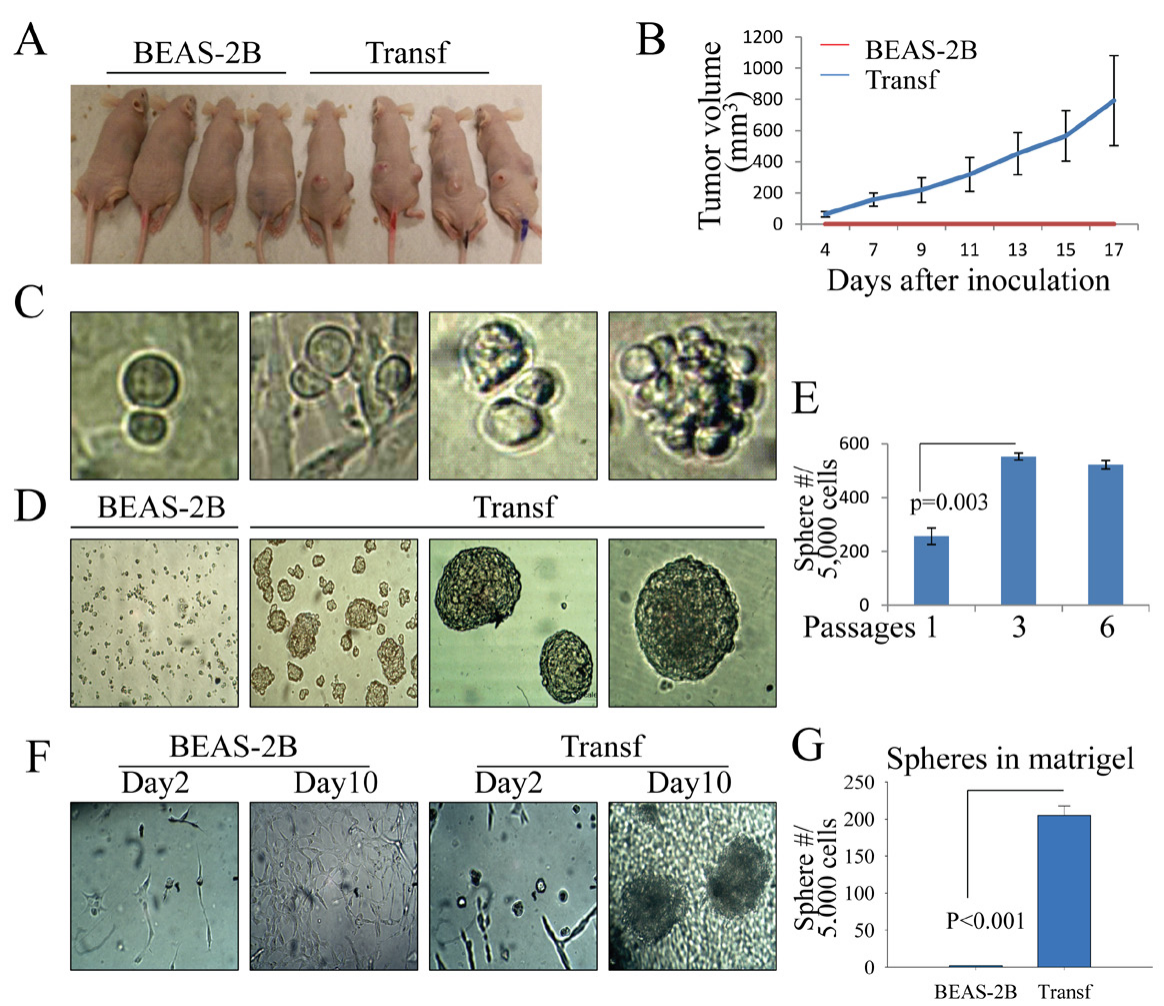
The transformed cells induced by As^3+^ have features of CSCs (A & B) Tumorigenicity of the parental (BEAS-2B) and transformed (Transf) cells was determined by injecting 1 × 10^6^ cells subcutaneously into the flank of 6-week-old male BALB/c nude mice. The image shows tumor sizes 17 days after injection. (C) Asymmetric division of the transformed cells. (D) Tumor sphere formation assay for the parental (BEAS-2B) and transformed (Transf) cells. (E) The sphere-forming cells were enriched after the passage of tumor spheres. (F) Three-dimensional (3D) culture of the parental (BEAS-2B) and transformed (Transf) cells in a Matrigel matrix. Tumor spheres were visible for the transformed cells 10 days after initial seeding of the cells in Matrigel matrix. (G) Quantification of the tumor spheres of the parental cells and the transformed cells in 3D culture.

We have previously shown that a consecutive low-concentration As^3+^ treatment, which induced a sub-lethal stress of the cells, for four to six months could induce the transformation of the human bronchial epithelial cell line BEAS-2B, as shown by the resistance of these cells to As^3+^-induced cell death and anchorage-independent growth in soft-agar [16]. Intriguingly, these transformed cells also exhibited a reduced ability to generate reactive oxygen species (ROS) in response to As^3+^. To expand these previous observations, we detailed the changes in morphology and tumorigenicity of these transformed cells. Despite continued culturing and passaging of the cells for several months, the parental cells that were cultured in As^3+^-free medium showed typical morphological features of epithelial cells, such as the cells were well-organized in sheets of cuboidal cells that were closely attached and exhibited an apico-basal polarity. In contrast, the transformed cells induced by As^3+^ showed fibroblast-like morphology with elongated, ramified and thin cell bodies that resembled mesenchymal cells or mesenchymal stem cells. Moreover, these transformed cells grew into multiple layers and formed sphere-shaped foci and demonstrated signs of loss of contact inhibition (data not shown). To test whether these transformed cells were malignant and tumorigenic *in vivo* in recipient mice, 1 × 10^6^ parental and transformed cells were separately inoculated into the nude mice subcutaneously. Tumor formation could be detected after as early as four days in all mice inoculated with the transformed cells. After 17 days of injection, the average diameter of tumors of the transformed cells rose to 1 cm. No single tumor was detected in the mice that were inoculated with the parental cells after 17 days (Figures 1A and 1B). Collectively, these results suggest that the transformed cells induced by the As^3+^-induced sub-lethal stress are highly tumorigenic.

### Transformed cells possess the characteristics of CSCs

During the routine culture and passage of the cells, we noted that many transformed cells generated two distinct daughter cells with different sizes after cytokinesis, which is indicative of the unequal distribution of cellular components into two daughter cells due to asymmetric division, a feature of the self-renewal of stem cells or CSCs [17] (Figure 1C). Closer monitoring of these unequally divided cells revealed that these cells are the major sources of forming sphere-shaped cell clusters (right panel, Figure 1C). To verify whether some of the transformed cells were possibly CSCs that were able to self-renew, we next transferred the parental cells and transformed cells into ultralow-attachment six-well plates containing tumorsphere formation medium. As depicted in Figure 1D, the transformed cells remained viable and formed tumorspheres after four to seven days of culture in serum-free medium. Some of the transformed cells formed giant spheres with a relatively smooth surface (Figure 1D, right two panels). In contrast, no viable cells or sphere-forming cells were observed among the parental cells (left panel, Figure 1D). To further determine the self-renewal capability of the sphere-forming cells, serial tumorsphere passage assays were performed. We found that the sphere-forming cells were enriched significantly through serial passage (Figure 1E). To test for another functional hallmark of self-renewal of the CSCs, we conducted 3D tumorsphere assays by seeding the cells in a Matrigel matrix to mimic the growth niche of CSCs. Again, the transformed cells, but not the parental cells, formed tumorspheres in this Matrigel matrix-based 3D culture (Figures 1F and 1G).

### The sphere-forming cells are tumorigenic in vivo

**Figure 2:**
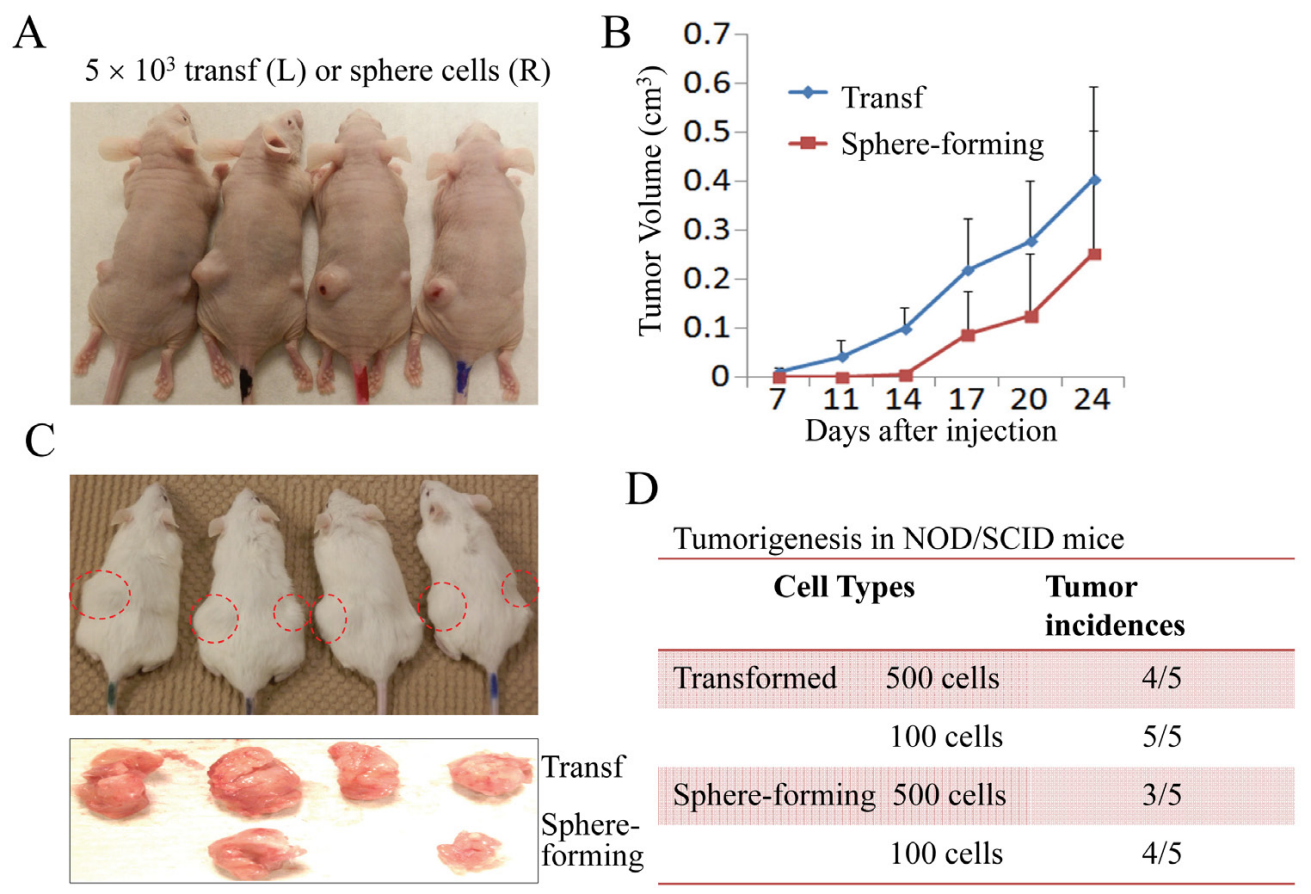
The transformed cells and sphere-forming cells are tumorigenic *in vivo* (A) The nude mice were subcutaneously injected with 5,000 transformed cells and sphere-forming cells at the left (L) and right (R) sides, respectively. (B) Tumor volumes were measured on the indicated days. (C) Tumorigenicity assay of the transformed cells and sphere-forming cells in NOD/SCID Il2rγ^−/−^ mice. (D) Tumor incidence rates of the transformed cells and sphere-forming cells in NOD/SCID mice after 45 days of injection with 100 or 500 cells.

The transformed cells induced by As^3+^ were highly tumorigenic in nude mice (Figures 1A and 1B). To determine whether the sphere-forming cells mentioned above were key contributors to the tumorigenicity of the transformed cells, we injected 10,000 transformed cells and sphere-forming cells into nude mice subcutaneously and compared the tumor growth rates of the transformed cells and the sphere-forming cells. The sphere-forming cells formed tumors, but they were smaller than those formed by the transformed cells (Figure 2A). Moreover, the tumor formation by the sphere-forming cells appeared to lag behind that of the transformed cells by approximately one week. Following that lagging period, a similar tumor growth rate was noted between the sphere-forming cells and the transformed cells (Figure 2B). To exclude the possibility that residual immunity, which provides an unfavorable niche for CSC growth in nude mice, might cause the delayed tumor occurrence of the sphere-forming cells, we next inoculated the cells in NOD/SCID Il2rγ^−/−^ recipient mice. Again, the sphere-forming cells formed smaller tumors than the transformed cells after two weeks of inoculation (Figure 2C). We speculated that this smaller tumor size and the delayed tumor occurrence of the sphere-forming cells in both nude mice and NOD/SCID mice might be a result of the resting state of the sphere-forming cells because these cells were cultured in a serum-free environment for one to two weeks before they were injected. An additional possibility is that this phenomenon was a reflection of the nature of the CSCs, since relative to the bulk tumor cells, CSCs are slower in cell cycle and proliferation. Indeed, when fewer cells (500 or 100 cells) were injected and monitored for 35 to 45 days in NOD/SCID mice, a similar tumor incidence rate was observed between the transformed cells and the sphere-forming cells (Figure 2D). These results suggest that the sphere-forming cells are the key transformed cells in tumorigenesis.

### Transformed cells express higher levels of stemness genes

**Figure 3:**
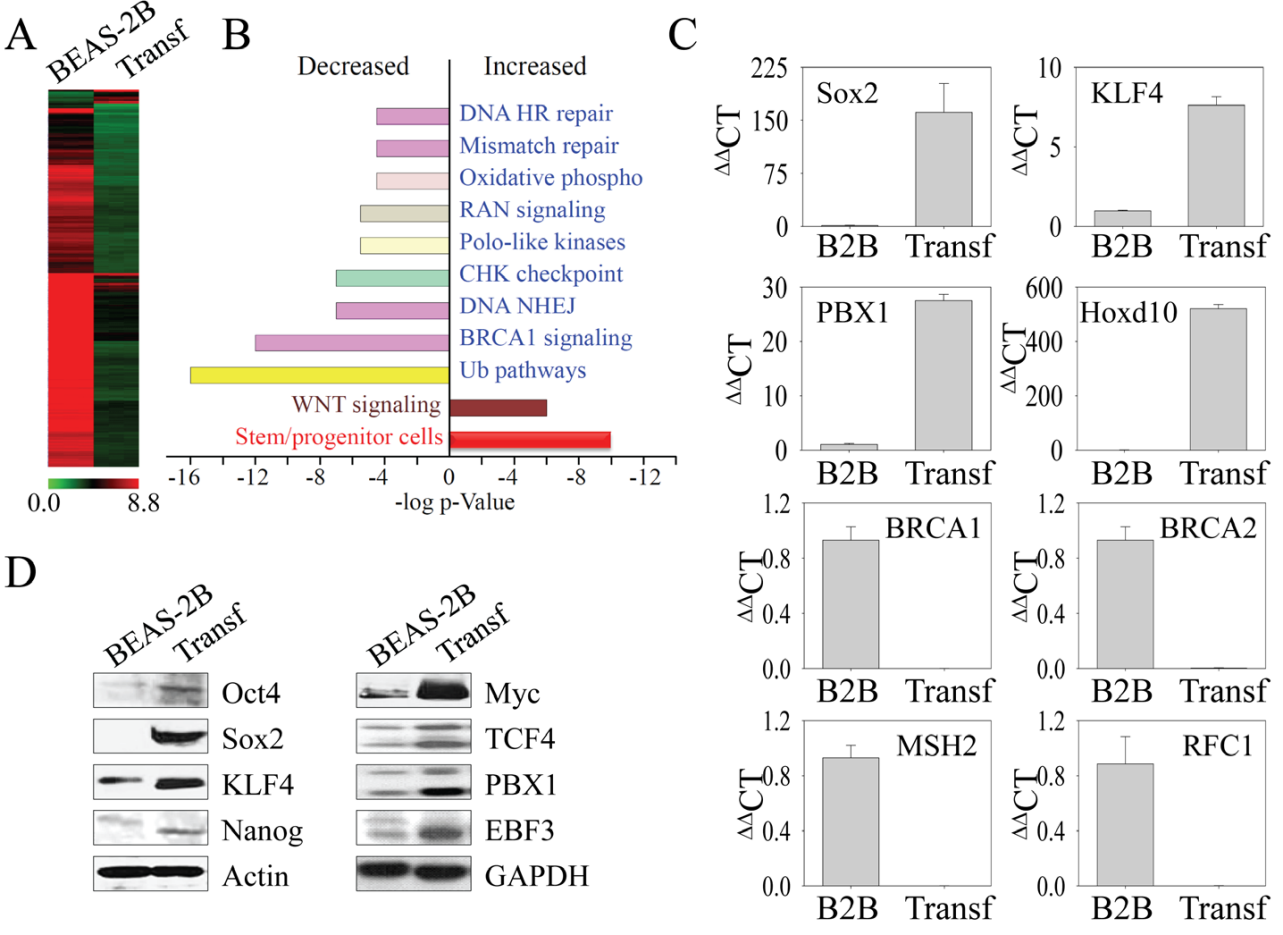
The transformed cells induced by As^3+^ express genes for the stemness of CSCs (A) Gene expression profiles were compared between parental (BEAS-2B) and the transformed (Transf) cells using Affymetric Human Gene 1.0 ST Arrays. (B) Ingenuity Pathway assay for the differentially expressed genes in the transformed cells compared to the parental cells based on BioRank Scores. (C) Verification of the differentially expressed genes by real-time PCR. (D) Western blotting to determine the expression levels of the transcription factors and proteins important for the self-renewal and pluripotency of the CSCs between parental (BEAS-2B) and the transformed (Transf) cells.

Increasing evidence suggests that some aggressive cancers or CSCs have gene expression profiles similar to embryonic stem cells (ESCs) and induce pluripotent stem cells (iPSCs) [18]. Furthermore, the state of the self-renewal of CSCs is established by the core regulatory circuitry of transcription factors for the pluripotency of the ESCs [19, 20]. Accordingly, we determined the mechanism underlying the key phenotypes, such as sphere-forming and tumorigenicity in NOD/SCID mice, of the transformed cells by comparing the global transcription profiles of the transformed cells to those of parental cells via microarray analysis. Unexpectedly, we found that a majority of genes are downregulated in the transformed cells (Figure 3A). We identified 227 genes that were upregulated and 2,369 genes that were downregulated more than 5-fold in the transformed cells compared to the parental cells. Gene ontology analyses revealed that the population of upregulated genes was enriched with genes associated with the stemness of stem cells or CSCs (Figure 3B) and the Wnt signaling pathway, whereas the downregulated genes were mostly associated with the protein ubiquitination and DNA repair pathways, such as DNA homologous recombination (HR), mismatch repair, and nonhomologous DNA end joining (NHEJ) (Figure 3B). Intriguingly, a group of genes important for the mitochondrial oxidative phosphorylation (OXPHOS) that linked to the generation of energy and ROS through the tricarboxylic acid cycle on the respiration chain was also highly down-regulated (Table 1). The expression of some of the key genes was validated by quantitative real-time RT-PCR, which confirmed that, consistent with the expression profiling by microarray analysis, genes such as Sox2, Klf4, PBX1, and Hoxd10 were upregulated, whereas those critical for DNA repair, such as BRCA1, BRCA2, MSH2, and RFC1, were downregulated in the transformed cells (Figure 3C).

**Table 1:** Decreased expression of the genes in oxidative phosphorylation in the sub-lethal stress-induced CSCs

ATP5B	−8.0
ATP5C1	−16.5
ATP5G1	−8.3
ATP5J2	−11.2
ATP5L	−2.0
ATP5O	−6.6
ATP6V0E1	−24.8
ATP6V1B2	−11.5
ATP6V1C1	−16.4
ATP6V1D (includes EG:299159)	−7.0
ATP6V1E1	−11.3
COX4I1	−14.9
COX6A1	−40.5
COX6C	−9.8
COX7B	−26.2
COX7C	−14.5
NDUFA2	−9.4
NDUFA9	−7.9
NDUFB5	−36.5
NDUFC2	−41.2
NDUFS5	−29.9
SDHB	−16.7
UQCR10	−7.2
UQCRC2	−12.9
UQCRQ	−20.9

To further address the observation that the transformed cells induced by As^3+^-induced sub-lethal stress have the properties of CSCs, the protein levels for the core regulatory circuitry of transcription factors for self-renewal and pluripotency of the CSCs were determined by Western blotting. In agreement with the data from the microarray and PCR, we noted an increased expression of Oct4, Sox2, Klf4, and Myc, the quartet of transcription factors essential for the maintenance of stemness of ESCs, iPSCs and CSCs, in the transformed cells (Figure 3D). In addition, we also observed elevated levels of TCF4, PBX1 and EBF3, which also contribute to the function of ESCs or CSCs, in the transformed cells. Nanog, another important transcription factor of the ESCs or CSCs, is marginally increased in the transformed cells.

### The CSCs in the transformed cells are CD61―

**Figure 4:**
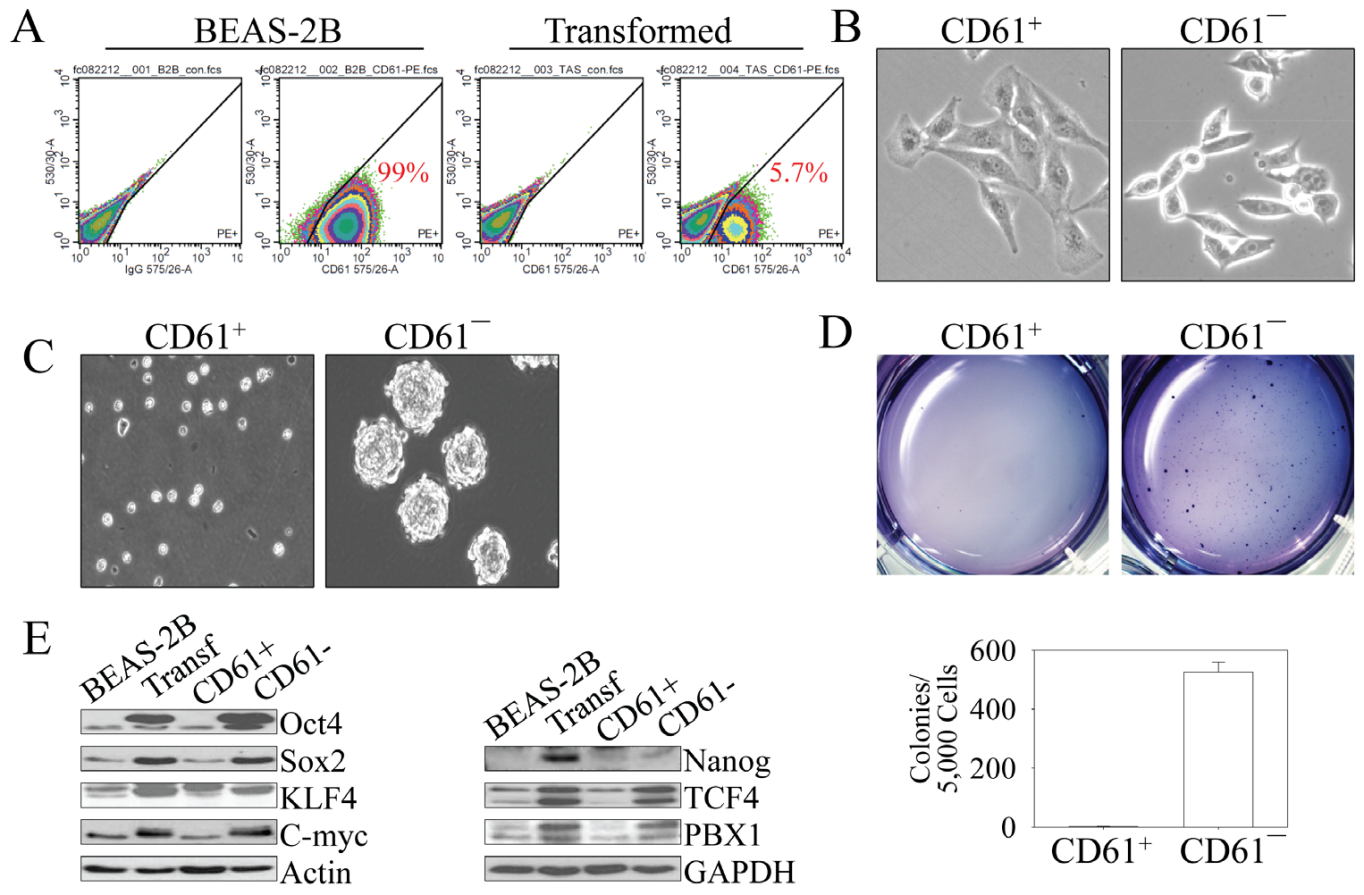
As^3+^-induced CSCs are CD61¯ (A) FACS analysis of the parental (BEAS-2B) and transformed (Transf) cells for the surface marker, CD61. The numbers inside the panels show the average percentage of the CD61^+^ cells. (B) Morphological difference between CD61^+^ cells and CD61¯ cells isolated from the transformed cell by FACS. (C) Capability of tumor sphere formation of the CD61^+^ cells and CD61¯ cells isolated from the transformed cell by FACS. (D) CD61¯ cells, but not the CD61^+^ cells, can form colonies in soft agar. Lower panel shows quantification of the colony formations by CD61^+^ cells and CD61¯ cells. (E) Immunoblotting for the determination of the expression levels of genes important for CSCs among the parental cells (BEAS-2B), transformed cells, CD61^+^ cells, and CD61¯ cells.

A consensus on CSC surface markers has not yet been reached. However, several markers, including CD133, CD24, CD44, CD166, EpCAM (CD326), CXCR4 (CD184), c-kit (CD117), and among others have been identified as surface markers of CSCs isolated from glioblastoma, breast cancer, pancreatic cancer, prostate cancer, or hepatocellular carcinoma [15]. To investigate potential CSC markers in the As^3+^-transformed cells, we conducted fluorescence-activated cell sorting (FACS) analysis for the expression of a panel of 15 stem/progenitor cell markers for the transformed cells. Except for CD49f and CD61, no other positive markers were detected (Figures 4A and data not shown). When we compared the expression of CD49f and CD61 between the parental cells and the transformed cells, we found that approximately 90-99% of both the parental cells and transformed cells are CD49f positive (data not shown). Thus, it is unlikely that CD49f is a CSC marker in the transformed cells. There is a significant difference in the percentage of CD61 positive cells between the parental cells and the transformed cells. Nearly 100% of the parental cells are CD61 positive (CD61^+^). In contrast, only about 6-10% of the transformed cells are CD61^+^ (Figure 4A), suggesting that more than 80% of the transformed cells are CD61^―^ cells. It is possible, thus, that these CD61^―^ cells are potentially the CSCs among the transformed cells. Examining the cell morphology showed that the CD61^+^ cells in the population of the transformed cells exhibited a morphology typical of epithelial cells, whereas the CD61¯ cells exhibited features of mesenchymal-like cells (Figure 4B). Other tests suggested that the CD61¯ cells are the cells capable of forming tumor spheres (Figure 4C) and colonies in soft-agar (Figure 4D). As an additional measure to support the hypothesis that the CD61¯ cells among the transformed cells that were induced by consecutive low-concentration As^3+^ treatments are CSCs, we evaluated the levels of the key transcription factors and functional proteins that are important for the stemness of CSCs. CD61¯ cells expressed higher levels of Oct4, Sox2, Klf4, Myc, TCF4, and PBX1 relative to the CD61^+^ cells or parental cells (Figure 4E). Despite the fact that the CD61^+^ cells in this experiment are isolated from the transformed cells through FACS, these cells showed a similar expression pattern of those selected markers to the non-transformed parental cells.

### The CD61¯ cell population is a prerequisite for tumor initiation *in vivo*

**Figure 5:**
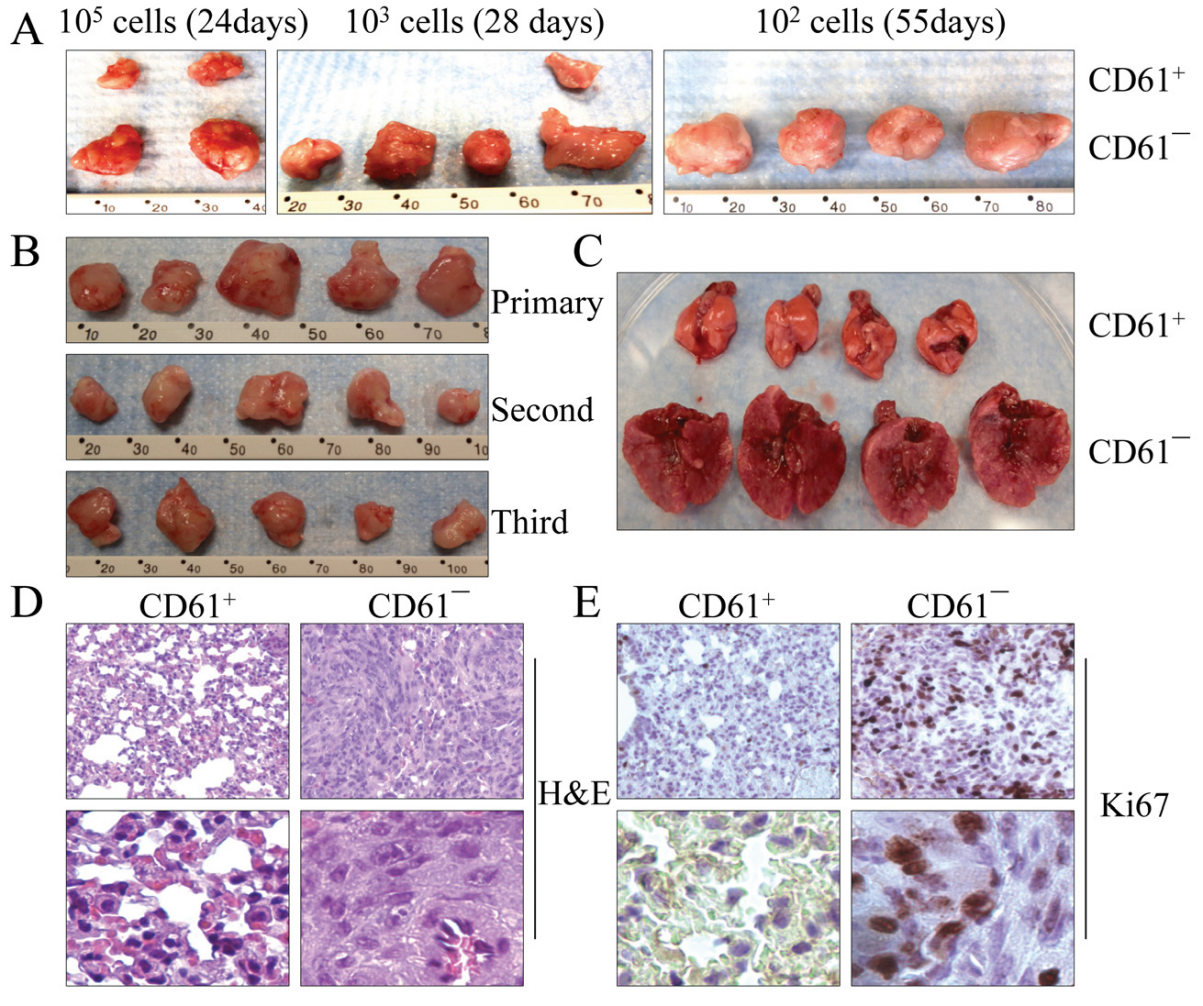
CD61¯ cells are able to self-renew *in vivo* (A) Limited dilution assay for the tumorigenicity of the CD61^+^ cells and CD61¯ cells in NOD/SCID Il2rγ^−/−^ mice. (B) Serial xenotransplantation of the CD61¯ cells in tumor formation in NOD/SCID Il2rγ^−/−^ mice. The data show the first, second and third generations of the tumors from serial xenotransplantation. (C) CD61¯ cells are highly metastatic to the lung after intravenous injection of 50,000 cells into the NOD/SCID mice. The mice injected with CD61^+^ cells and CD61¯ cells were euthanized 13 days after injections and their lungs were dissected for image documentation. (D) Histological images of lung tissues from the NOD/SCID mice received intravenous injection of the CD61^+^ cells and CD61¯ cells. Lower panels display magnified images from different fields of the original ones. (E) Cancerous lung tissues from the NOD/SCID mice received intravenous injection of the CD61¯ cells showed signs of high proliferation as determined by Ki67 staining. Lower panels display magnified image from different fields of the original ones.

To compare the tumorigenic capability of the transformed cells with different CD61 statuses, a limited dilution assay was performed. Initially, we injected 100,000 CD61^+^ and CD61^―^ cells to the left and right flanks of NOD/SCID Il2rγ^/−^ mice, respectively. At 24 days after injection, tumor volume was measured. The volume of the CD61¯ cell-derived tumor was approximately 4- to 5-fold larger than the tumor derived from the CD61^+^ cells (Figure 5A, left panel). To further confirm this observation, we reduced the number of cells to 1,000 in this assay and monitored tumor growth for 28 days. All 4 sites injected with CD61¯ cells developed a tumor. In contrast, only 1 out of 4 sites injected with CD61^+^ cells had a tumor, which had a much smaller size (Figure 5A, middle panel). When 100 cells were inoculated at 55 days after the injection, tumor mass was found only in the injection sites of CD61^―^ cells (Figure 5A, right panel). No tumor was found in the injected sites of CD61^+^ cells throughout the observation period.

One of the primary characteristics of CSCs is the ability to self-renew *in vivo*, which allows them to reconstitute tumors identical to the original tumor in animals through serial xenotransplantations. To test the self-renewal characteristic, the primary tumors from the CD61¯ cells were dissociated to create single-cell suspensions. One hundred CD61¯ cells that were isolated by FACS from the tumors were serially transplanted to secondary NOD/SCID Il2rγ^/−^ mice. As indicated in Figure 5B, the CD61¯ cells from the parental xenografted tumors were able to recapitulate the original CD61¯ cells and form the second and third generations of the tumors, indicating that the CD61¯ cells are truly CSCs that have an *in vivo* self-renewing capability.

### CD61¯ CSCs are highly metastatic to the lung

CSCs are thought to be responsible for long-term tumor growth and relapse. These cells have also been viewed as the major source of metastasis of the tumors from the original sites to distant organs. To determine whether the CD61¯ CSCs are metastatic, we directly introduced 50,000 CD61^+^ and CD61¯ cells into NOD/SCID Il2rγ^−/−^ mice by tail vein injection, respectively. The mice receiving CD61¯ cells showed severe shortness of breath 10 days after injection and had to be euthanized on the 13th day after injection. The lungs of the mice injected with CD61¯ cells were 3- to 4-fold larger than the lungs of the mice injected with CD61^+^ cells (Figure 5C). Multiple nodules of different sizes could be observed on surface and interior lung tissues from the mice injected with CD61¯ cells. Pathological and histological examinations revealed a colonized growth of cancerous tissues within the parenchyma of these lungs (Figure 5D). Ki67 staining confirmed a high proliferation of the cancer cells in these cancerous lesions (Figure 5E). No abnormality was observed in the lungs from the mice injected with CD61^+^ cells. These data clearly suggested that the CD61¯ CSCs are highly metastatic to the lung. Except lung metastasis, no other metastatic signs were noted in the mice injected with CD61¯ cells, possibly because of the short period of observation.

### JNK-dependent Myc expression is central to the As3+ sub-lethal stress-induced CSCs

**Figure 6:**
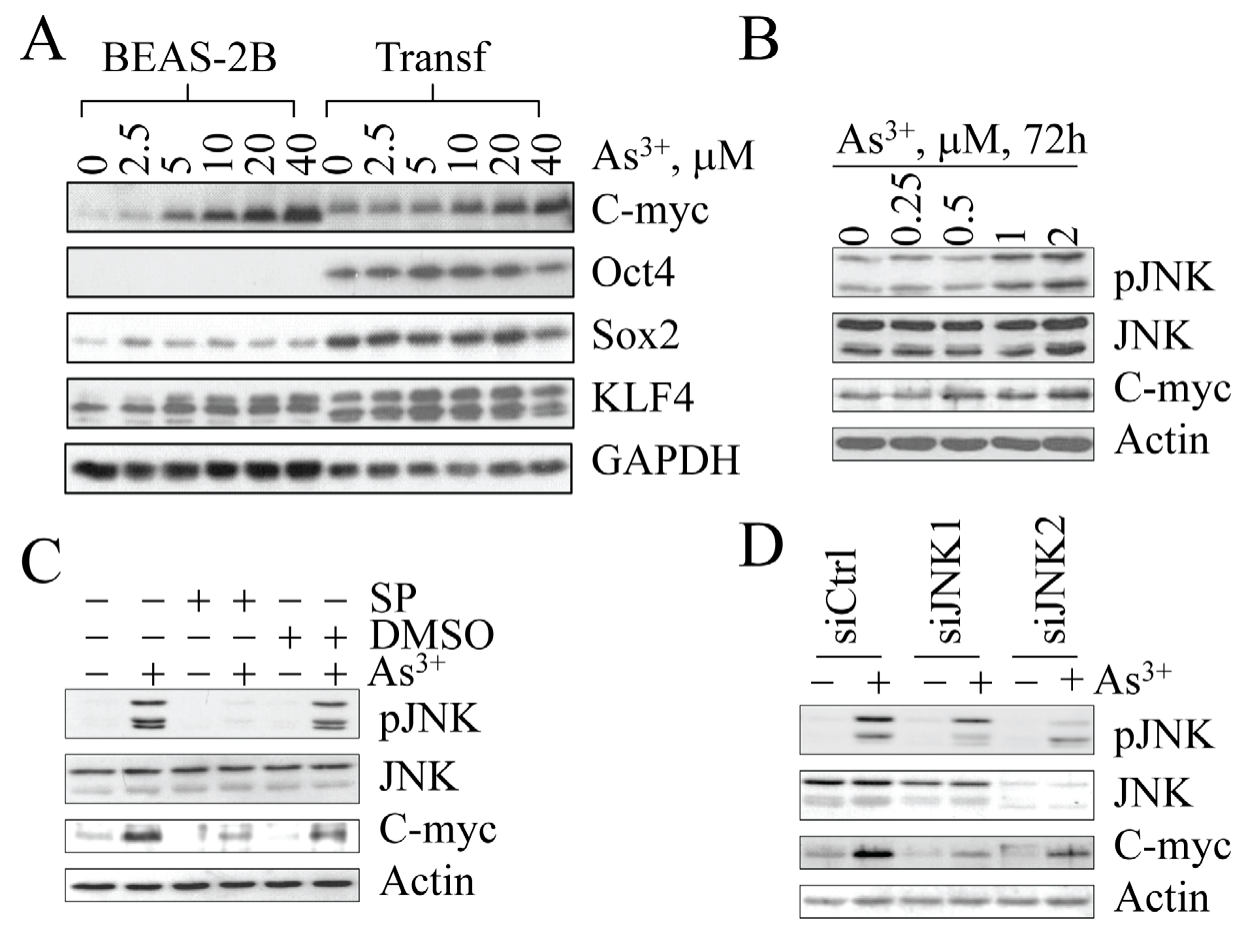
As^3+^-induced Myc expression is JNK-dependent (A) Dose-dependent induction of Myc protein by As^3+^. As^3+^ is unable to induce Oct4, Sox2 and Klf4 in both the parental cells (BEAS-2B) and the transformed cells (Transf). (B) Myc induction in the parental cells is correlated with JNK activation in response to lower concentrations of As^3+^ for 72 h. (C) Inhibition of JNK by JNK inhibitor SP600125 (SP) prevented Myc induction by As^3+^. (D) Silencing JNK by siRNAs specific to JNK1 or JNK2 inhibited As^3+^-induced Myc expression.

Overwhelming evidence suggests that the expression of Oct4, Sox2, Klf4, and Myc is the key for the maintenance of ESCs, adult stem cells and CSCs. Enforced overexpression of these quartet factors has been shown to be sufficient to reprogram differentiated somatic cells into iPSCs. We were interested in how sub-lethal stress induced by consecutive low concentration As^3+^ treatments of the cells triggers the formation of CSCs without the introduction of any of the exogenous quartet factors. For our initial studies, we focused on the possibility that As^3+^ activates Oct4, Sox2, Klf4, and Myc, which are the essential transcription factors for reprogramming, self-renewal and pluripotency. To test this hypothesis, we treated the parental cells, among which approximately 99% of the cells are CD61^+^, and the transformed cells, among which approximately 90% of the cells are CD61¯, with various concentrations of As^3+^ for 6 h. Analogous to our earlier experiments indicated in Figures 3 and 4, under basal conditions, increased expression of the quartet factors can be observed in the transformed cells (Figure 6A). In the dose-dependence study, only Myc can be induced by As^3+^ in both parental cells and the transformed cells. No conclusive effect of As^3+^ on Oct4, Sox2 and Klf4 was observed in either parental cells or the transformed cells (Figure 6A).

Our previous reports showed that As^3+^ is a potent activator of JNK [21, 22], a protein kinase that has been linked to Myc expression [23]. To mimic the cell transformation procedure in which the cells were in sub-lethal stress condition induced by constitutive low-concentration As^3+^ treatment, we treated the cells with 0.25 to 2 μM As^3+^ for 72 h and then measured JNK activation along with Myc expression. As shown in Figure 6B, the activation of JNK by As^3+^ is correlated with Myc expression. The role of JNK on As^3+^-induced Myc expression was further confirmed by inhibition of JNK activity through either JNK inhibitor or siRNAs specifically targeting JNK1 or JNK2. As shown in Figure 6C, JNK inhibitor, SP600125, attenuated the level of Myc protein significantly in the cells treated with As^3+^ (Figure 6C). In addition, As^3+^-induced Myc expression was reduced in the cells where JNK1 or JNK2 was silenced by siRNAs (Figure 6D).

### CD61low lung cancer correlates with the poorer survival of the patients after tumor resection

**Figure 7:**
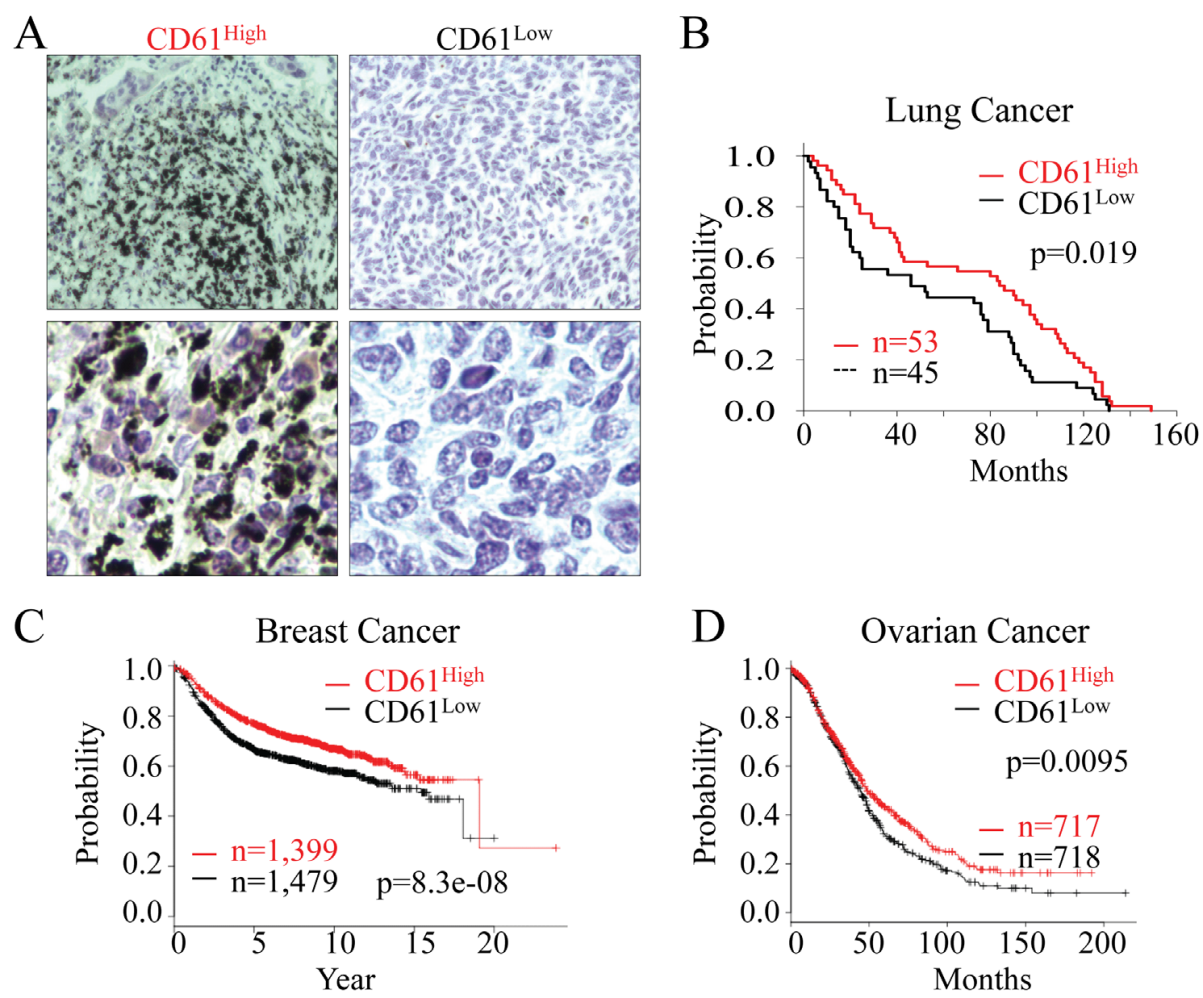
CD61 status predicts the overall survival of the cancer patients (A) The levels of CD61 expression in human lung cancer tissues were determined by mmunohistochemistry staining of the lung cancer tissues in tissue microarray with the anti-CD61 antibody. The lung cancers were stratified into CD61^high^ and CD61^low^ lung cancers based on the strength of CD61 staining on tissue array slides that contain 100 human lung cancer samples. Lower panels display the magnification of the original images in a different field. (B) Kaplan-Meier survival probability of the lung cancer patients based on the CD61 status in the lung cancer tissues. (C) Relapse-free survival probability of the breast cancer patients based on the expression of the CD61 gene. (D) Overall survival probability of the ovarian cancer patients based on the expression of the CD61 gene.

The fact that CD61¯ cells have the characteristics of CSCs, are highly tumorigenic and are metastatic to the lung raised the possibility that CD61 status might be important in the prognosis of patients with lung cancer and other cancers. To explore whether there is a correlation between the CD61 status and patient survival, we performed an immunohistochemical analysis on panels of a tissue microarray containing 100 archived human lung cancer samples of different stages and histological types using a CD61 antibody. Based on the strength of CD61 staining, we arbitrarily stratified human lung cancers into a CD61 high group (CD61^high^) and a CD61 low group (CD61^low^) (Figure 7A). We found no significant correlation between CD61^high^ or CD61^low^ and lung cancer types or stages (data not shown). However, as shown in Figure 7B, the status of CD61 is a strong predictor of patient survival. The CD61^low^ was associated with shorter survival time of the patient after tumor resection (p = 0.019 based on log-rank test for trend). To gain additional insights into the prognostic value of CD61, we also searched the Kaplan-Meier plotter database (www.kmplot.com) [24], focusing on the CD61 gene expression level and the relapse-free survival of breast cancer and overall survival of ovarian cancer patients. Among the available data for total of 2,878 breast cancer patients, a lower expression of CD61 (1,479 patients) is a strong predictor of poorer relapse-free survival with a p-value of 8.3e-08 (Figure 7C). The same conclusion was reached for the overall survival of 1,435 ovarian cancer patients, among which 718 patients are CD61^low^, although the statistical significance, p = 0.0095 is not as strong as that of the breast cancer patients (Figure 7D). Together, these data clearly indicate that a subset of human cancers have CD61^low^ status that serves as an important predictor of survival for patients with lung cancer, breast cancer, and ovarian cancer.

## DISCUSSION

By consecutive treatments of the human bronchial epithelial cells with low concentrations of As^3+^ for six months, which induced a sub-lethal stress of the cells, we revealed reprogramming of these cells to form CD61¯ CSCs without the enforced expression of the exogenous transcription factors essential for self-renewal and pluripotency. In addition to the higher tumorigenicity and metastasis as evident in studies of limited dilution, serial xenotransplantation and intravenous injection of the cells in NOD/SCID Il2rγ-^/−^ recipient mice, we also demonstrated that CD61 status is a prognostic factor for predicting the overall survival of the lung cancer patients as well as the patients with breast cancer or ovarian cancer. Furthermore, our approaches of gene expression profiling and ontology assays delineate some important and new features of the CD61¯ CSCs, most notably, is the decreased expression of genes that are prominent in several DNA repair pathways and oxidative phosphorylation (Fig. 3 and Table 1).

As^3+^ is a well-known environmental human carcinogen that is able to activate a number of intracellular protein kinases and transcription factors that are involved in cell growth, cell cycle, pro- or anti-apoptosis, and malignant transformation [4, 5]. The current standard of As^3+^ in drinking water established by both the World Health Organization (WHO) and the Environmental Protection Agency (EPA) of the United States of American is less than 10 ppb (0.13 μM). However, more than one hundred million people worldwide are still at risk for exposure to drinking water with levels of As^3+^ exceeding 100 to 1,000 ppb (1.3 - 13 μM) [3]. A number of epidemiology and case-control studies demonstrated a strong association of environmental As^3+^ exposure, either through drinking water contamination or air pollution, to the increased incidence rate of human lung cancer [25]. By analyzing As^3+^ levels in soils and the data of cancer registries and statistics, recent studies by Putila and Guo revealed a correlation between the sediment levels of As^3+^ and human lung cancer, and concluded that soil As^3+^ contributes to more then 5,000 lung cancer cases annually in the US [26]. Mechanistically, As^3+^ was believed to be able to activate several oncogenic intracellular signaling pathways that mediate malignant transformation or carcinogenesis of the normal cells induced by As^3+^ [27, 28]. It remains to be fully elucidated, however, whether CSCs are involved in the As^3+^-induced lung carcinogenesis.

The capability of As^3+^ in inducing CSCs was first implicated in studies using *in utero* As^3+^ exposure in mice by Waalkes and colleagues [29, 30], which showed that fetal exposure to As^3+^ enhanced the susceptibility and aggressiveness of skin tumors with an increased number of CD34^+^ cells, the tentative CSCs. It was speculated that this enhancement in skin carcinogenesis was resulted from the effects of As^3+^ on embryonic stem cells and/or keratinocyte stem cells. This assumption was compensatorily confirmed by the fact that As^3+^ could convert non-transformed prostate epithelial stem/progenitor cells and renal stem cells into the CSCs [6, 7, 31, 32]. An additional evidence to support this notion is from “whole-life” As^3+^ exposure, which showed that parental exposure to As^3+^ prior to breeding, during pregnancy and lactation increased tumor formations in the offsprings [33]. A unique feature of these tumors in offsprings, esp., for the lung adenocarcinoma and hepatocellular carcinomas, is the overabundance of the CSCs in these tumors.

The present report showed that As^3+^ can reprogram non-stem cells into the CSCs. The compelling question to be asked is how As^3+^, without assistance from the overexpression of any of the required transcription factors, activates a reprogramming process from a program that normally promotes differentiation. Our biochemical data indicated that As^3+^ appears to be unable to regulate Oct4, Sox2 and Klf4 in both parental cells and the transformed cells (Figure 6A), despite an increased expression of Oct4, Sox2 and Klf4 in the transformed cells induced by As^3+^. However, As^3+^ is highly capable of inducing Myc expression in the parental cells as well as the transformed cells (Figure 6A). Disruption of the JNK signaling by either JNK inhibitor SP600125 or JNK specific siRNAs prevented Myc induction by As^3+^ (Figures 6C and 6D). Thus, it is very likely that sustained Myc induction by As^3+^ sub-lethal stress plays central role in the formation of CSCs.

Although the CSCs induced by As^3+^ showed increased expression of the genes important for the pluripotent and self-renewal of the CSCs, it is unexpected that the expression of the majority of the genes is down-regulated. Among these down-regulated genes, the most important groups of genes are those involved in DNA repair and OXPHOS (Fig. 3 and Table 1). At the present, the DNA damage responses and repair capability in stem cells or CSCs are poorly characterized. The finding of massive down-regulation of the DNA repair genes may implicate the nature of genomic instability of the CSCs. In human breast epithelial cells and breast CSCs, it was found that increased expression or activity of the EZH2 protein, a key subunit of the Polycomb Repression Complex 2 (PRC2) that induces trimethylation of the lysine 27 on histone H3, repressed most of the Rad51 paralog proteins important for DNA HR repair [34, 35]. This is possibly true for the As^3+^-induced CD61¯ CSCs, because an increased expression of EZH2 protein was noted in these cells (Chang et al, unpublished observation).

Some earlier studies suggested that stem cells or CSCs are incapable of using OXPHOS for energy metabolism due to poor development of the mitochondria or decoupling of the respiratory chain in mitochondria [36]. This notion can be supported by the observed down-regulation of the genes in OXPHOS in the As^3+^-induced CD61¯ CSCs in the present report (Table 1). Under physiological conditions, cells use OXPHOS in mitochondria for complete oxidation of glucose to generate ATP as well as ROS. In OXPHOS, glucose is metabolized to pyruvate that is channeled to tricarboxylic acid (TCA) cycle for the maximal ATP production. However, stem cells or CSCs are mostly resided in a hypoxic niche that favors glucose metabolism through aerobic glycolysis (also known as Warburg effect) to generate lactate in cytoplasm. Thus, decrease of ROS in a given population of cells is indicative of repressed OXPHOS and enhanced glycolysis. The glycolysis and OXPHOS of the glucose homeostasis are reciprocally regulated. For example, the ROS generated from OXPHOS can inactivate the key glycolysis enzymes, whereas NADPH generated from the pentose phosphate pathway (PPP) of the glycolysis is a powerful antioxidant against ROS. It is well-known that ROS are potent inducers of tumor suppressor p53 that can inhibit the activities of the stemness genes, such as Oct4, c-myc, Sox2, Klf4, and Nanog [37]. In addition, higher level of ROS may directly oxidize and inactivate these stemness transcription factors. Furthermore, ROS are highly capable of inducing DNA damage, differentiation and apoptosis of the CSCs. Thus, low concentration of ROS is critical for self renewal of CSCs by reducing the sensitivity of CSCs to oxidative stress-induced DNA damage and apoptosis. Many reports indicated that the pluripotent status of stem cells or CSCs rely on glycolysis [38, 39]. Several oncogenic growth signals, such as, Hedgehog, STAT3, Akt, and c-Myc, can promote glycolysis by inducing expression or activity of the rate-limiting enzymes in the glycolysis pathway that primes or supports the stemness status induced by Oct4, Myc, Sox2, and Klf4 [40, 41].

A number of studies indicate that there is a hierarchical organization of tumor cells, in which CSCs are at the apex and responsible for generating differentiated progeny to repopulate cancer cells with high heterogeneity [15]. Despite this, whether all cancers are driven by CSCs is still a topic of intensive debate. A major hurdle currently facing in CSC studies is the inconsistence of CSC surface markers among several different types of cancers. Although some surface markers, including CD133, CD24, CD34, CD44, CD166, and EpCAM, have proven to be useful for the isolation of subsets enriched for CSCs in multiple types of tumors [14], a considerable number of reports failed to detect these markers on certain subpopulations of CSCs. Consistent with this notion, we were also unable to detect these tentative markers on the As^3+^-induced CSCs. However, we defined a specific CD61¯ CSC population based on the expression of the stem cell transcription factors, mesenchymal morphology, tumorigenicity, and metastasis to the lung in NOD/SCID Il2rγ^−/−^ mice (Figures 4-5). CD61 has been previously identified as a bona fide marker for the enrichment of CSCs from HER2-induced breast cancer in mice [42]. A heterogeneous expression of CD61 among a number of human breast cancer cell lines has been recently reported [43]. In the present study, we found that nearly 100% of the parental cells and 6% of the transformed cells are CD61^+^. Several tests clearly indicated that the CD61^+^ cells are neither tumorigenic nor CSCs. In contrast, the CD61¯ cells possess CSC features *in vitro* and *in vivo*.

As^3+^ is one of the major non-tobacco carcinogens associated with lung cancer of never smokers [44]. The findings from the present study provide novel mechanistic insights into lung carcinogenesis due to environmental exposure to As^3+^ or other carcinogens. It has been widely believed that an enforced introduction of the exogenous quartet of transcription factors, including Oct4, Sox2, Klf4, and Myc, is essential for the establishment of iPSCs or CSCs from somatic cells. Most recent studies, however, also revealed that mildly acidic conditions can induce iPSCs from somatic cells, indicating that some sub-lethal stimuli may able to trigger reprogramming without assistance from the overexpression of any of the required transcription factors [45, 46]. Our findings that sub-lethal stress induced by consecutive low concentration of As^3+^ treatment induces CD61¯ CSCs provided the first evidence to support such notion. It is unclear, at the present, whether the same mechanisms are involved in the reprogramming of the cells in response to mildly acidic conditions and As^3+^-induced sub-lethal stress. Additional biochemical tests in our studies revealed that As^3+^ is unable to regulate Oct4, Sox2 and Klf4 in both parental cells and the CSC-like cells or the CD61¯ CSCs (Figure 6A), despite an increased expression of Oct4, Sox2 and Klf4 in the latter cells induced by As^3+^. However, sub-lethal stress induced by As^3+^ is highly capable of inducing Myc protein in the parental cells as well as CD61¯ CSCs (Figure 6B). Thus, it is very likely that sustained Myc induction by As^3+^-induced sub-lethal stress plays a central role in the formation of CSCs. It is worth to test whether this is true in other sub-lethal stress-induced generation of the iPSCs or CSCs.

## MATERIALS AND METHODS

### Cell culture and treatment

The human bronchial epithelial cell line BEAS-2B was purchased from the American Type Culture Collection (ATCC, Manassas, VA) and cultured in DMEM with 10% FBS. For As^3+^ treatment, BEAS-2B cells were treated with 0.25 μM NaAsO_2_ continuously for six months. Cells cultured without As^3+^ treatment for six months were used as parental cell control in all experiments. Cell transformation was evaluated by using *in vitro* soft agar colony-forming assay and *in vivo* tumorigenicity experiment in athymic nude mice. For the soft agar colony-forming assay, 1% agar was melted and mixed with 2 × DMEM medium in a 1:1 ratio to produce a supporting layer (0.5%) in a 6-well tissue culture plate (1 ml/well). The bottom agar layer was allowed to solidify at room temperature for 20 min. The top layer containing 0.3% agar (1 ml/well) was prepared by mixing stock agar solutions with 5,000 cells in 2× DMEM medium and was laid on the top of the bottom agar layer. Fresh DMEM medium was added every 3 days. Three weeks later, colonies were stained with a 0.05% crystal violet solution, counted, and photographed under a microscope.

### Clonal formation assay

For holoclone assays, parental or transformed cells were plated at a clonal density of 10 cells/well in 6-well plate in DMEM medium with 10% FBS. Seven days later, the holoclone numbers were counted using a microscope.

### Flow cytometry analysis and fluorescence-activated cell sorting (FACS)

The cells were dissociated, and one million individual cells were resuspended in 100 μl of sorting buffer (PBS containing 0.5% FBS, 2 mM EDTA) and then stained with 20 μl of the indicated antibodies conjugated with PE or FITC purchased from BD Biosciences (San Jose, CA) for 30 min at 4°C. Antibodies against human immunoglobulin conjugated to PE or FITC (BD) were used as antibody isotype controls. The cells were washed in PBS and centrifuged at 800×g for 2 min twice and then resuspended in 500 μl sorting buffer. FACS analysis was performed using CellQuest software (BD Biosciences).The cells were sorted on the FACS Vantage SE/SORP with a Diva flow cytometer (BD Biosciences).

### In vitro Tumor sphere formation

The self-renewal capability of the transformed cells was determined by an *in vitro* tumor sphere formation assays. Briefly, 5,000 cells were seeded onto ultralow-attachment 6-well plates (Corning life sciences, Tewksbury MA) in serum-free DMEM/F12 (Invitrogen, Grand Island, NY) medium containing 1× B27 supplement (Invitrogen), 20 ng/ml EGF (Invitrogen) and 20 ng/ml bFGF (Invitrogen). Fresh medium was added every three days for two weeks. Tumor spheres were counted and photographed using a microscope. For tumor sphere passage assays, the primary spheres were dissociated and reseeded in ultralow-attachment 6-well plates for the second round of tumor sphere formation. All experiments were performed in triplicate for each condition and were repeated at least three times.

### Western blot analysis

Cells were lysed in 1× RIPA lysis buffer containing protease and phosphatase inhibitors on ice. Each sample, containing 40 μg of protein, was resolved on a 4-12% Bis-Tris Gel with MOPs Running Buffer and transferred to PVDF membranes. The blots were then probed with various antibodies. Horseradish peroxidase (HRP)-conjugated secondary antibodies were used to detect the specific immunoreactions. Blots were developed with an enhanced chemiluminescence regent (Pierce) and exposed to X-ray film. The following primary antibodies were used: Oct4, Sox2, Klf4, c-Myc, Nanog, TCF4, PBX1, EBF3, CD44, CD49f, E-Cad, KRT5, Fibronectin, p63, Vimentin, β-catenin, EZH2, pEZH2, JNK, pJNK, Stat3, pStat3, Akt, pAkt, Smad3, pSmad3^s208^, Actin, and GAPDH. All antibodies were purchased from Cell Signaling Technology (Danvers, MA), Abcam (Cambridge, MA), or Santa Cruz Biotechnology, INC. (Santa Cruz, CA).

### Gene expression profiling and real-time PCR

Total RNA was extracted from 1×10^7^ parental cells, As^3+^-induced transformed cells and tumor sphere-forming cells using TRIzol reagent (Invitrogen) and dissolved in diethyl pyrocarbonate-treated H_2_O to a final concentration of 500 ng/μl. RNA integrity was determined by electrophoresis. Gene expression profiling was performed using the Human Gene 1.0 ST Array (Affymetrix). To identify differentially expressed genes between the parental cells, transformed cells and tumor sphere-forming cells, data were uploaded to Partek® software (version 6.6 Copyright © 2012 Partek Inc., St. Louis, MO) using the Exon workflow. Quantile normalization, log2-transformation, probeset summarization (median polish), and background subtraction (RMA) were performed on the data upon import. Exons were summarized to genes by averaging exon expression across each gene. Differential expression was calculated for each comparison by performing an analysis of variance (ANOVA). Genes with a change of at least ± 1.5-fold and an FDR-corrected p-value < 0.05 are reported. Hierarchical clustering (Euclidean Distance and Average linkage clustering) was performed using the TM4 Microarray Software Suite's MultiExperiment Viewer V4.8. The gene expression profiling data were validated by quantitative real-time PCR, as reported previously.

### Tumorigenicity assay in nude mice

For the evaluation of tumorigenicity, 1 × 10^6^ parental or transformed cells were injected subcutaneously into the flank of 6-week-old male BALB/c nude mice purchased from the Jackson Laboratory (Bar Harbor, ME). Tumor size was measured on the indicated days with calipers, and tumor volume was calculated based on the formula (width^2^ × length)/2. All animal procedures were preapproved by the Institutional Animal and Care Use Committee (IACUC) of the Wayne State University.

### Transplantation assays using CSCs in NOD/SCID Il2r-/- mice

To evaluate the tumorigenicity of the putative CSCs, aliquots of 100 or 500 parental cells, transformed cells, CD61^+^ cells, or CD61¯ cells were injected subcutaneously into the flank of 6-week-old male NOD/SCID Il2r^−/−^ mice purchased from Jackson Laboratory (Bar Harbor, ME). The mice were euthanized on the indicated days, followed by the removal of tumors. Tumor size was measured by volume, as calculated based on (width^2^ × length)/2, or by direct weighting. For the serial xenotransplantation of the CSCs, the primary tumors or the secondary tumors that were removed from NOD/SCID mice were dissociated mechanically and enzymatically to generate single cell suspensions for a subsequent injection into the new NOD/SCID mice.

### Statistical analysis

In general, all data are shown as the mean ± standard deviation. Statistical significance in differences was determined by either SigmaPlot 11 software or unpaired two-tailed Student's *t-*tests. In all analyses, a p*<*0.05 was considered statistically significance.
